# Cyclin-Dependent Kinase Inhibitors and Their Therapeutic Potential in Colorectal Cancer Treatment

**DOI:** 10.3389/fphar.2021.757120

**Published:** 2021-12-21

**Authors:** Oana-Maria Thoma, Markus F. Neurath, Maximilian J. Waldner

**Affiliations:** ^1^ Department of Medicine 1, Friedrich-Alexander-Universität Erlangen-Nürnberg, Erlangen, Germany; ^2^ German Center for Immunotherapy (DZI), University Hospital Erlangen, Friedrich-Alexander-Universität Erlangen-Nürnberg, Erlangen, Germany; ^3^ Erlangen Graduate School in Advanced Optical Technologies (SAOT), Friedrich-Alexander-Universität Erlangen-Nürnberg, Erlangen, Germany

**Keywords:** cyclin-dependent kinases (CDKs), CDK inhibitors, CDK4/6 cell cycle inhibitors, colorectal cancer, CRC therapy, cell cycle

## Abstract

Cyclin-dependent kinases (CDKs) are key players in cell cycle regulation. So far, more than ten CDKs have been described. Their direct interaction with cyclins allow progression through G1 phase, transitions to S and G2 phase and finally through mitosis (M). While CDK activation is important in cell renewal, its aberrant expression can lead to the development of malignant tumor cells. Dysregulations in CDK pathways are often encountered in various types of cancer, including all gastrointestinal (GI) tract tumors. This prompted the development of CDK inhibitors as novel therapies for cancer. Currently, CDK inhibitors such as CDK4/6 inhibitors are used in pre-clinical studies for cancer treatment. In this review, we will focus on the therapeutic role of various CDK inhibitors in colorectal cancer, with a special focus on the CDK4/6 inhibitors.

## Cyclin-Dependent Kinases and Their Role in Cell Cycle Progression

Cell cycle is defined as the process through which the cell replicates all its genomic material and divides into two identical cells (Alberts et al., 2002). It consists of four phases: gap 1 (G1), where the cell grows in size and transcribes the RNA and protein necessary during cell division; synthesis or S phase, where all chromosomes are being replicated; gap 2 (G2), where cell growth and protein synthesis continue; and mitosis or M phase, where the cell restructures its membrane and organizes the newly synthesized chromosomes and then divides into two daughter cells. Before entering cell cycle, highly proliferative cells such as stem cells and lymphocytes are in a reversible cell cycle arrest, known as quiescence or gap 0 (G0). However, other cells such as neurons or adipocytes are irreversibly arrested in G0 phase, a phenomenon often described as cellular senescence. Senescence is also predominant in highly damaged cells, acting as a protective mechanism during the DNA damage response (DDR) (Terzi et al., 2016).

Each cell cycle phase, as well as transitions from one phase to the other, are tightly regulated by interactions between cyclins and cyclin-dependent kinases (CDKs) (Johnson and Walker, 1999). In general, cyclins directly bind CDKs and induce the formation of cyclin—CDK complexes. This promotes CDK activity and therefore ensures activation of specific transcriptional programs that allow cell cycle progression. More than ten CDKs are known to be involved in various events during cell cycle. From these, CDK1, 2, 3, 4, and 6 directly mediate cell cycle progression.

Transition from quiescence or G0 phase in G1 phase is modulated by growth factor signals or mitogenic stimulation. These result in the upregulation of Cyclin D, which binds to and activates CDK4 and CDK6 to promote cell commitment to enter G1 phase (Jinno et al., 1999; Lea et al., 2003). High CDK4/6 expression and activation ensures cell progression through G1 phase (Mende et al., 2015; Topacio et al., 2019).

On the molecular level, CDK4 and 6 phosphorylate Retinoblastoma (Rb) and promote the accumulation of E2F, a direct regulator of genes necessary during DNA synthesis. Furthermore, CDK4 and CDK6 activation initiates cell growth through activation of mammalian target of rapamycin complex 1 (mTORC1) (Romero-Pozuelo et al., 2020). Besides, CDK4 and 6 are involved in the control of DNA replication mechanisms (Braden et al., 2008). Along with CDK4/6, CDK2 and CDK3 are also activated during G1 phase. Rb phosphorylation, and therefore the accumulation of E2F during G1 phase, directly mediate the upregulation of Cyclin E in late G1 phase, which binds and activates CDK2. Formation of CDK2/Cyclin E complex maintains Rb phosphorylated in order to promote G1/S phase transition (Massague 2004; Horiuchi et al., 2012). However, CDK3 upregulation during late G1 phase seems to be independent of Cyclin D, E or A binding (Braun et al., 1998). Interestingly, the upregulation of CDK2 has been also shown to be important during the G1/S checkpoint in response to DNA damage. For example, knocking-down CDK2 in the HCT116 tumor cell line significantly reduced p53 phosphorylation in response to hydroxyurea (HU) and suppressed G1/S cell cycle arrest (Bacevic et al., 2017). Some recent studies also described a role of CDK2 directly after mitosis, as an intermediate level will remain in the cells that continue proliferating, while those that lack CDK2 can enter quiescence or so called gap 0 (G0) (Spencer et al., 2013; Gookin et al., 2017). On the other hand, high levels of Cyclin C/CDK3 have been reported to directly mediate quiescence (Ren and Rollins 2004).

The beginning of S phase is marked by increasing levels of Cyclin A, which binds CDK2. The complex formed by Cyclin A/CDK2 drives the cells through S phase and promotes DNA replication. During late S/G2 phase, increased levels of Cyclin A induce CDK1 activation, which drives entry into mitosis (Gavet and Pines, 2010; Kalous et al., 2020). Later, the formation of CDK1/Cyclin B complex triggers progression through M phase. Along with its important role in successful cell mitosis (Vassilev et al., 2006), CDK1 can also influence the remodeling of cell adhesion complexes during G1, S and G2 cell cycle phases (Jones et al., 2018) and promotes protein synthesis during proliferation (Haneke et al., 2020). Interestingly, CDK1 is reported to be the only necessary cyclin-dependent kinase during cell cycle, being able to bind to all cyclins and drive all events during cell division (Santamaria et al., 2007).

Several other CDKs are known to be involved in cell cycle progression as well. CDK7, for example, is an important cell cycle regulator. Its binding to Cyclin H and mating-type 1 protein (Mat1) induces the formation of CDK-activating kinase (CAK) complex. CAK activity is crucial to promote CDK2 and CDK1 binding to cyclins, therefore allowing cell division (Fisher and Morgan, 1994; Larochelle et al., 2007; Olson et al., 2019). CDK5 upregulation is mostly observed in, but not limited to, neurons, and is often correlated to cell apoptosis. Nevertheless, it can also regulate the cell cycle by phosphorylating Rb and interacting with E2F during G1 phase (Zhang et al., 2010; Chang et al., 2012; Futatsugi et al., 2012). CDK8 is a partner of Cyclin C and its expression has been shown to be important in stabilizing Cyclin C activity during cell cycle (Tassan et al., 1995; Barette et al., 2001). Interestingly, CDK8 and Cyclin C, as well as CDK19/Cyclin C complex, are strongly required during p53-dependent p21 transcriptional activation, for cell cycle arrest in response to DNA damage (Donner et al., 2007; Audetat et al., 2017). Last, cyclin-dependent kinases such as CDK9 and CDK13 are not directly controlling cell cycle phase transitions, but are rather involved in transcription mechanisms, by associating with Cyclin T or Cyclin K (Garriga et al., 2003; Yu et al., 2010; Greifenberg et al., 2016).

**FIGURE 1:**
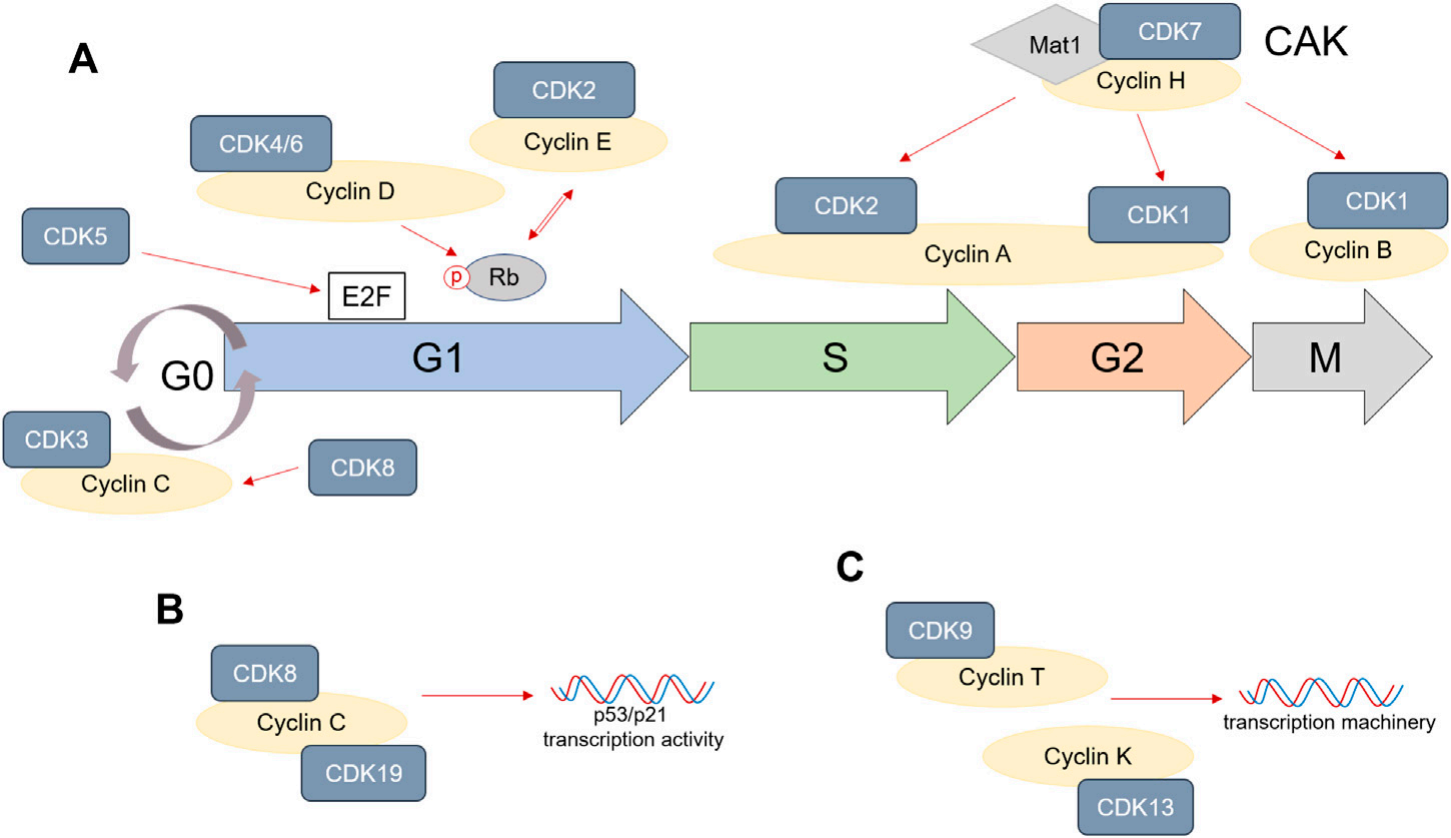
Cyclins and cyclin-dependent kinase (CDK) role in cell cycle. **(A)** CDK1, 2, 3, 4/6, and 7 are directly involved in progression through cell cycle phases by associating themselves with various Cyclins. CDK5 can have a direct impact on E2F accumulation, especially in cancer, while CDK8 activation stabilizes Cyclin C. **(B,C)** CDK8, 9, 13, and 19 are not directly involved in cell cycle progression, but are involved in either p53/p21 transcription (CDK8/19) or the DNA transcription machinery (CDK9/13).

To summarize, entry into cell cycle depends on mitogenic or growth factor signals. CDK4/6/Cyclin D complex formation promotes Rb phosphorylation and accumulation of free E2F, which ensures progression through G1 phase. CDK5 activity also increases E2F levels during G1. High levels of E2F during late G1 induce CDK2/Cyclin E complex that in return further phosphorylates Rb and promotes G1/S transition. At the beginning of S phase, Cyclin E levels decrease and CDK2 forms a complex with the increasing Cyclin A, which not only ensures progression through S phase, but also transition into G2 phase. CDK2/Cyclin A complex is especially regulated by the CDK7/Cyclin H/Mat1 complex, also described as CAK. CAK also regulates CDK1/Cyclin A complex formation during late G2 and Cyclin B binding to CDK1 during mitosis. Any disturbances to the cell cycle machinery will result in cell cycle arrest. CDK2 and CDK3 are especially important in mediating either quiescence or senescence. Indirectly, CDK8, 9, 13, and 19 also mediate cell cycle, being involved in the transcription machinery, while CDK5 can directly modulate apoptosis as well. A schematic representation of the important role of CDKs in cell cycle is shown in Figure 1. While normal cells are able to activate the necessary mechanisms for cell cycle arrest when the DNA is damaged, these pathways are usually suppressed or non-existent in tumor cells, enabling them to continue progression through cell cycle. The following sections will address the CDK’s role in the tumor cell division and how therapies targeting CDKs can modulate CRC development.

## Cyclin-Dependent Kinase Expression in Human Colorectal Cancer

Changes in the regulatory mechanisms that control cell division are often related to accumulation of mutations and/or epigenetic dysregulations of cancer related genes and can contribute to the molecular mechanisms of colorectal cancer (CRC). CRC tissue often shows changes in genes related to cell cycle arrest (p16 and p21), apoptosis (p53) or proliferation (PCNA) (Yue et al., 2003; Kruschewski et al., 2011). Multiple other mutations have also been described to be involved in CRC development. As a result, CDKs expression can be changed in tumor cells.

When looking at the signature of differentially expressed genes (DEGs) in patients with CRC compared to normal colon tissue, an upregulation in CDK1 gene expression is often observed (Zhao et al., 2019; Ding et al., 2020; Li et al., 2020). Interestingly, the expression of CDK1 in the nucleus and cytoplasm has been used as a marker to describe patterns in the overall survival of patients with CRC (Sung et al., 2014). Staining of over 164 cancer samples from primary CRC revealed that CDK1 is expressed in both cell nucleus and cytoplasm to a certain degree. The evaluation of nuclear/cytoplasm (N/C) ratio on these samples showed that high N/C expression is often found in patients with overall worse survival and a N/C > 1.5 can be considered a risk factor. Furthermore, high CDK1 expression is predominant in patients with resistance to 5-fluorouracil (5-Fu), a common CRC treatment, and it seems to reduce the effect of chemotherapy (Zhu et al., 2020). An upregulation of CDK1 in CRC has been also observed in response to other drugs such as: betaxol, penbutolol and propofol amongst others (Mastrogamvraki and Zaravinos, 2020).

CDK2, 4 and 6 levels in CRC are closely related to the Rb protein hyperphosphorylation, which seems to promote cancer progression. CDK4/6 is usually amplified in colon tumors compared to healthy epithelium (Mastrogamvraki and Zaravinos, 2020; Jardim et al., 2021). Abundant levels of CDK4 are especially observed in CRC patients with enhanced dysplasia and are correlated to increased tumor cell proliferation (Zhang et al., 1997; Bartkova et al., 2001). Some CDK2 expression is normally found in healthy epithelium. However, its upregulation can be predominantly observed human CRC tissue samples (Yamamoto et al., 1995). Interestingly, CDK2 overexpression in primary CRC tumors is also linked to lymph nodes metastasis, but not liver metastasis (Li et al., 2001; McCurdy et al., 2017). Nevertheless, a certain CDK2 activity has been reported to improve recurrence-free survival (RFS) of patients after surgery (Yamamoto et al., 1995). A similar pattern to CDK2 expression in CRC is observed in CDK3 levels as well. Its overexpression has been linked to metastasis and tumor cell invasion, where it seems to be promoting epithelial to mesenchymal transitions (Lu et al., 2016).

CDK5 expression is also reported to be much higher in CRC cells compared to normal epithelium and it correlates to increased tumor growth and poor prognosis (Zhuang et al., 2016; de Porras et al., 2019). Most important, CDK5 is directly involved in the degradation of the cell cycle inhibitor p21 and can enhance CDK2 activity, which might further promote tumor cell growth (Huang et al., 2016). Decreased survival rates are also observed in CRC patients with high CDK9 and CDK13 levels (Kim et al., 2012; Wang et al., 2019). Interestingly, high CDK9 expression in CRC tissue was negatively correlated with cytotoxic CD8^+^ T cell infiltration. Furthermore, these infiltrated cells showed increased cell exhaustion in CDK9-high tumors, which might further affect patient outcome (Wang et al., 2019). Last, CDK8 overexpression in CRC is also considered as a marker for poor patient prognosis, being directly linked to β-catenin activation amongst others and therefore promoting cancer growth (Firestein et al., 2008; Firestein et al., 2010; Seo et al., 2010). Overall, cyclin-dependent kinase activation is often observed in colorectal cancer and seems to promote tumor progression and an overall worse survival of patients, as summarized in Table 1.

**TABLE 1:** Effects of increased CDK expression in patients with colorectal cancer.

Gene	Expression in CRC	Patient outcome	References
CDK1	Upregulated in tumor tissue compared to normal tissue	Decreased overall patient survival	Ding et al. (2020)
	Ratio between nuclear and cytoplasmatic expression can be used as an indicator of patient outcome	Interferes with 5-Fu therapy	Sung et al. (2014)
	Medication can further upregulate CDK1 in CRC		Zhu et al. (2020)
			Mastrogamvraki and Zaravinos (2020)
CDK2	A normal CDK2 expression is also found in healthy colon	Increased expression in normal colon tissue after surgery is correlated to a good prognosis	Yamamoto et al. (1995)
	Upregulated in CRC tissue compared to normal tissue		Li et al. (2001)
	Overexpression correlated to lymph node metastasis		McCurdy et al. (2017)
CDK3	No expression found in normal colonic tissue	Not described	Lu et al. (2016)
	Overexpressed in CRC tissue and metastatic tissue		
CDK4/6	Upregulated in CRC samples compared to healthy tissue	Poor prognosis in patients with strong CDK4 expression in tumors	Jardim et al. (2021)
			Mastrogamvraki and Zaravinos (2020)
			Zhao et al. (2003)
CDK5	Upregulated in tumor tissue compared to the adjacent healthy tissue	Increased tumor growth	de Porras et al. (2019)
	Can upregulate CDK2 expression as well	Poor patient prognosis	Zhuang et al. (2016)
			Huang et al. (2016)
CDK8	Overexpressed in CRC tissue compared to matched healthy tissue	Promotes cancer growth	Firestein et al. (2010)
		Poor patient prognosis	Seo et al. (2010)
CDK9/13	High in CRC tissue	Worse overall patient survival	Kim et al. (2012)
			Wang et al. (2019)

## The Functional Role of CDKs in CRC

Basic research using murine knockout models or *in vitro* gene silencing in tumor colon cancer cell lines also provided some understanding for the relevance on CDKs in CRC development. Since CDKs are vital components of the cell cycle, creating knockout mouse models is usually unsuccessful. This is because most CDKs (e.g. CDK1, 4, 6, 9, and 13) are critical during embryonic development, as is summarized in (Campbell et al., 2020). Similarly, conditional knockout models often show severe impairments.

Nevertheless, some fundamental research data in regards to the role of CDKs in colorectal cancer are available. For example, it is know that CDK4 activation in CDK4^R24C/R24C^Apc^+/min^ mice leads to significant increased in tumor vascularity in comparison to CDK4^+/+^Apc^+/min^ mice or APC^+/min^ mice (Abedin et al., 2010), while knocking out CDK4 in APC^+/min^ mice reduces adenoma development (Karim et al., 2013). CDK5 silencing via transfection can directly reduce the proliferation of human HCT116 and SW480 tumor cell lines (Zhuang et al., 2016). Similarly, knocking down CDK9 in HCT116 and HT29 tumor cell lines induced their apoptosis by Caspase 7 cleavage (Rahaman et al., 2019). Furthermore, it reduced Cyclin D1 protein expression, suggesting cell cycle arrest induction in these cells.

Stable silencing of CDK8 and CDK19 in Colo205 human colon cancer cells reduced β-catenin/TCF-dependent transcription (Dale et al., 2015). A direct link between CDK8 and β-catenin regulation in tumor cell proliferation and death has also been described, where inactivation of CDK8 by siRNA transfection in HCT116 cells significantly reduced the RNA and protein levels of β-catenin (He et al., 2011). Generally, silencing CDK genes in colon cancer cells reduces their proliferation and induces cell death, which makes them an attractive target for the development of new inhibitory therapies.

## CDK Inhibitors as a Potential CRC Treatment

CDK inhibitors are also often used in basic research to understand molecular mechanisms of CDK activation in cell cycle regulation or tumor cell proliferation. This section describes the current understanding on the potential use of various CDK inhibitors to mediate colorectal cancer development.

### CDK7-Specific Inhibitors

Samuraciclib and SY-1365 are inhibitors of CDK7 activity. Interestingly, the colon cell line HCT116 is particulary sensitive to Samuraciclib, which induces their apoptosis and cell cycle arrest (Patel et al., 2018). Its mechanism of action is mostly based on inhibition of phosphorylation of CDK7 substrates like CDK1 and 2. One important advantage of Samuraciclib is its availability as an oral drug that can accumulate at the tumor site upon multiple doses, as shown by the *in vivo* HCT116 murine tumor xenograf model. CDK7 inhibition was also successful when using SY-1365, in more than 26 types of cancer types, including colon cell lines (Hu et al., 2019).

### CDK1/2-Specific Inhibitors

SU9516 and CVT-313 are known to directly inhibit CDK2 activity. The use of SU9516 for *in vitro* treatment of HT29, RKO and SW480 human colon carcinoma cell lines revealed that it can successfully induce their apoptosis and cell cycle arrest (Lane et al., 2001; Yu et al., 2002). CDK2 inhibition also significantly decreases free E2F, but increases E2F/Rb complexes, therefore arresting the tumor cells. This effect was dependent on the duration of the treatment, since more E3F/Rb complexes were observed after 48 h than after 24 h in HT29 cell line. Inhibition of CDK2 in patient-derived human cell lines using CVT-313 has minimal effect on cell death (Somarelli et al., 2020). Nevertheless, combined therapy using CDK2 and 9 inhibitors significantly increased the numbers of cells arrested in G2/M.

RO-3306 is a CDK1-specific inhibitor can be used to induce apoptosis in a specific type of BRAF-mutated colorectal cancer cells (Zhang et al., 2018). Interestingly, this inhibitor induced Caspase 8-regulated cell death when combined with the MEK inhibitor, cobimetinib, while most CDK inhibitors promote apoptosis via Caspase 3 cleavage.

### CDK5, 8/19, and 9-Specific Inhibitors

CP668863 or 20-223 is a CDK5 inhibitor whose cytotoxic potential has been evaluated in CRC settings as well (Robb et al., 2018). Interestingly, 20-223 is 65-fold more potent for cell growth inhibition than the pan CDK inhibitor AT7519. Its cytotoxicity has been evaluated on SW620, DLD1 and HT29 tumor cell lines. 20-223 also significantly inhibited tumor growth in xenograf models and reduced the migration of colon cancer cells, which shows its potential for CRC therapy.

The development of MSC2530818 was fine tuned to specifically inhibit CDK8/19 (Czodrowski et al., 2016). This compound can be orally administered and it is well tolerated by mice. Treatment with MSC2530818 of mice subjected to an *in vivo* xenograft model using SW620 human colon cell line showed its potential to reduce tumor growth. CDK8/19 inhibition by MSC2530818 it is known to directly reduce STAT1 phosphorylation, further proving its efficacy.

CDKI-73 is a potent CDK9 inhibitor, which shows increased cytotoxicity against the HT29 and HCT116 human carcinoma cell lines (Rahaman et al., 2019). *In vitro* treatment of these cell lines revealed that CDKI-73 reduces the expression of survival genes. Its effect has also been tested in *in vivo* HT116 xenograf models. CDKI-73 significantly reduced tumor growth without being over toxic to the mice.

### Purvanalol and Roscovitine

Purvanalol and Roscovitine (Celiciclib or CYC202) are common CDK inhibitors effective against CDK2, 4, and 5 activity. Purvanalol is known to induce apoptosis and autophagy of HCT116 colon tumor cells by activating endoplasmatic reticulum (ER) stress (Coker-Gurkan et al., 2015). Its effect is nevertheless limited to wildtype HCT116, while Bax-deficient HCT116 cells are resistant against this treatment. This effect can be overcome by combining of Purvanalol with 3-MA, an inhibitor of autophagy, which promotes Purvanalol-induced apoptosis in Bax−/− HCT116 as well (Coker-Gurkan et al., 2014). Roscovitine has a similar effect on apoptosis induction in HCT116 tumor cells, but on a weaker scale than Purvanalol (Gurkan et al., 2013; Coker-Gurkan et al., 2015). Analysis of Roscovitine-induced apoptosis using Raman spectroscopy revealed changes in amide I and III bands, common of protein and DNA alterations (Akyuz et al., 2011). HCT116 cell death in presence of Roscovitine has been shown to be enhanced during polyamine depletion or phosphatase nuclear targeting subunit (PNUTS) knockdown (De Leon et al., 2010; Arisan et al., 2012). More important, the effect of Roscovitine is especially higher in combination to current chemotherapeutic drugs such as 5-Fu or doxorubicine, as shown by the experiments done with SW48, SW116 and SW837 colon cancer cell lines (Abaza et al., 2008).

### Wogonin

Wogonin is a flavone isolated from Scutellaria baicalensis known to inhibit CDK2, 4, 8, and 9. Nevertheless, its effect is not specific to only CDKs, but it also downregulates activation of PI3K/Akt and Stat3 signaling pathways (Wang et al., 2014; Tan et al., 2019). Along with its role in inducing apoptosis and autophagy of colorectal tumor cells, Wogonin can also induce cell cycle arrest in both G1 and G2/M cell cycle phases (He et al., 2013; Tan et al., 2019). Interestingly, Wogonin treatment of wildtype mice subjected to AOM/DSS tumor model reduces tumor growth by facilitating nuclear translocation of tumor suppresor p53 (Feng et al., 2018).

### Flavopiridol

Flavopiridol or Alvocidib is effective in inhibiting most CDKs: CDK1, 2, CDK4/6 and 9, by inducing cell cycle arrest and apoptosis of human colon tumor cell lines (Sausville et al., 2000; Kim et al., 2003; Okada et al., 2017). Treatment of CRC cell lines with flavopiridol enhances cell death when used in combination with chemotherapeutic agent gemcitabine or γ-radiation (Jung et al., 2001; Jung et al., 2003). Furthermore, a combination of docetaxal, flavopiridol and 5-Fu is described to more effective in inhibiting tumor growth and inducing increased apoptosis in HCT116 tumor cells, than any of the drugs alone (Guo et al., 2006). Phase I and phase II studies in patients with untreated advanced colorectal cancer showed little efficacy and was terminated early (Aklilu et al., 2003). Overall, it appears that Flavopiridol works best when coupled with other chemotherapeutic drugs.

### Other Pan CDK Inhibitors

Along with Purvanalol, Roscovitine, Wogonin and Flavopiridol, various other molecules have been described to inhibit multiple CDKs. For example, AT7519 is able to inhibit CDK1, 2, 4/6, and 9 and therefore induce colon cancer cell death. Its potency has been observed in xenograf mouse models using HCT116 and HT29, where tumor regression was observed upon multiple doses (Squires et al., 2009). Nevertheless, other CDK inhibitors such as 20-223 seem to be more effective than AT7519 (Robb et al., 2018). Pan CDK inhibitor AG-012986 has been shown to significantly reduce the colony formation of HCT116 colon carcinoma in a concentration-dependent manner, by inducing arrest into G1 phase (Zhang et al., 2008). Indirubin derivates are also known to reduce proliferation of DLD1 and HT29 tumor cell lines (Kim et al., 2009). Last, SNS-032 or BMS-387032, a specific inhibitor against CDK2, 7, and 9, was used to significantly reduce the intestinal tumor burden of Ink4/Arf-null Min mice (Boquoi et al., 2009). All in all, these data provide important insight on the effectiveness of CDK inhibitors in colorectal cancer therapy.

## CDK4/6 Inhibitors Use in CRC

**FIGURE 2:**
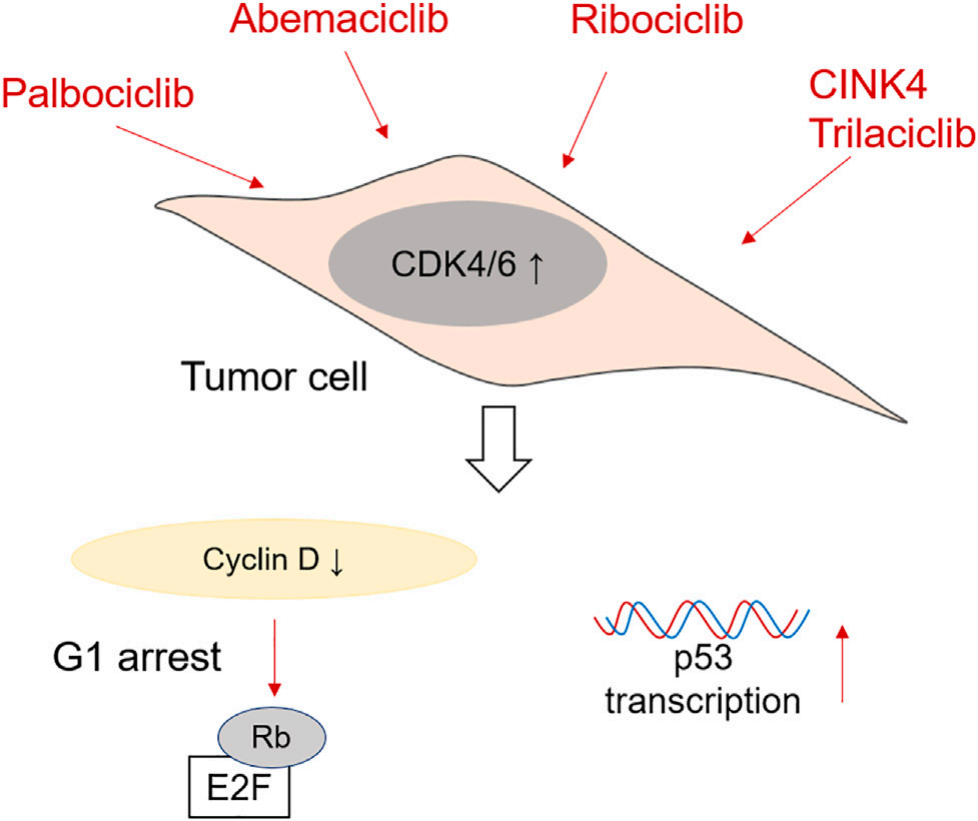
Mechanism of action of CDK4/6 inhibitors in CRC cells. Palbociclib, Ademaciclib, Ribociclib, CINK4 and Trilaciclib are able to prevent the formation of Cyclin D/CDK4/6 complexes, which reduces Retinoblastoma phosphorylation and induces G1 cell cycle arrest. Palbociclib has been shown to be effective in promoting p53 transcription after irradiation as well.

When thinking about preventing cell cycle progression and proliferation of tumor cells, CDK4/6 inhibitors are very efficient. The most commonly used are Ribociclib, Palbociclib, Abemaciclib and Trilaciclib. CDK4/6 inhibitors are especially effective at treating breast cancer amongst others, many of them being nowadays tested in phase I and II clinical trials (Wu et al., 2020). Nevertheless, they are also being tested as therapy for colorectal cancer. A schematic representation of the mechanism of action of CDK4/6 inhibitors is shown in Figure 2.

### CINK4 and Trilaciclib

Small molecule CINK4 is a triaminopyrimidine derivative specially designed to inhibit the activity of CDK4 in tumor cells. *In vitro* treatment of HCT116 colon tumor cell line with CINK4 prevented their cell growth by reducing Cyclin D/CDK4 complexes and Rb phosphorylation (Soni et al., 2001). Furthermore, intraperitoneal injection of CINK4 every 12 h was successful in reducing tumor growth in an *in vivo* mouse xenograf model using HCT116 tumor cells. Trilaciclib (CoselaTM) is known to directly induce reversible G1 cell cycle arrest and inhibit the formation of complexes between CDK4/6 and Cyclin D. As of 2021, Trilaciclib is used in a multinational trial (ClinicalTrials.gov Identifier: NCT04607668 in United States) in treating microsatelite stable metastatic CRC, in patients treated with FOLFOXIRI and Bevacizumab (Dhillon 2021). This clinical study has been recently approved and is at the moment recruiting participants in USA, Europe (Hungary, Italy, Poland, Slovakia, Spain, Ukraine, United Kingdom) and China.

### Abemaciclib

Patients with advanced and metastatic breast cancer can be treated with the CDK4/6 inhibitor Abemaciclib (also known as LY2835219, Verzenio, Verzenios, Ramiven). This inhibitor is also involved in various clinical trials for treating other advanced solid tumors such as melanoma or lung cancer (Shapiro et al., 2013; Fujiwara et al., 2016). The potential of Abemaciclib to treat colorectal cancer has been tested in mice with human tumor xenographs using Colo205 and A375 (Tate et al., 2014). The mice were treated orally in a concentration-dependent manner. The authors suggest that a constant level of 200 ng/ml Abemaciclib in plasma are necessary to arrest the tumor cells in G1 phase, as shown by Rb phosphorylation data. This shows that treatment using multiple doses might promote tumor cell cycle arrest in humans as well. Indeed Abemaciclib therapy in CRC patient cohort during a clinical trial induced stable disease even in a patient with KRAS and p53 mutated tumor cells (Patnaik et al., 2016). At the moment, Abemaciclib, in combination with LY3214996 (ERK1/2 inhibitor) and Cetuximab (EGFR inhibitor), is undergoing evaluation in Phase I and Phase II clinical trials in patients with metastatic CRC (ClinicalTrials.gov Identifier: NCT04616183). Recruiting phase is set to be completed in December 2021.

### Palbociclib

The efficacy of Palbociclib (PD-0332991) in inhibiting CDK4/6 activity has been assessed in human colon carcinoma cell lines as well (Li et al., 2014). Palbociclib successfully arrested various tumor cells (HT29, Colo205 and DLD1 amongst others) in G1 cell cycle phase, by reducing the phosphorylation of Rb. Interestingly, its therapeutic effect does depend on Rb presence (Heijink et al., 2011). Nevertheless, *in vivo* administration of Palbociclib in ApcMin mice successfully reduced tumor cell proliferation without affecting normal epithelial cells. It is very important to remark that Palbociclib mechanism of action directly targets the transcriptional activity of p53 after exposure to radiation and therefore, its efficacy might be limited to p53-expressing CRC tumors (Fernandez-Aroca et al., 2019). Palbociclib is also involved in a phase II clinical trial (ClinicalTrials.gov Identifier: NCT03981614), where it is used in combination with chemotherapeutic drug TAS-102 for KRAS/NRAS metastatic or unresectable CRC. First phase of the study has been recently completed (June 2021), but no data are momentarily available.

### Ribociclib

Treatment of HT29 and SW480 colon tumor cell lines with Ribociclib (or LEE011) significantly decreases their viability and induces G1 cell cycle arrest in concentration dependent manner (Lin et al., 2020). Similarly to the other CDK4/6 inhibitors, Ribociclib also reduces the phosphorylation of Retinoblastoma protein. Furthermore, used in combination with 5-FU, it increases significantly p53 phosphorylation. Ribociclib treatment was also used in a study case on a young female diagnosed with desmoid tumors (DT) (Santti et al., 2019). She underwent colectomy and various other surgeries to remove the tumors, as well as irradiation therapy. Unfortunately, the treatment with cytotoxic drugs usually used to treat these cancers did not reduced the tumors. The addition of Ribociclib, together with goserelin and letrozole therapy, stabilized temporarily the tumors and gave symptomatic relief. A Phase I clinical trial for treating selected malignancies, including CRC, using Ribociclib in combination with TNO155 (SPH2 inhibitor) is currently running (ClinicalTrials.gov Identifier: NCT04000529). Patients are still being recruited in this clinical trial.

## Future Perspectives in CDK4/6 Inhibitor Therapy in CRC

There is no doubt that targeting cell cycle machinery, and especially cyclin-dependent kinase activity of tumor cells, offers new opportunities to treat patients with advanced colorectal cancer. Nevertheless, cancer itself is a multifactorial disease and therefore the treatment with just one drug is not always successful.

CDK4/6 inhibitor therapy in particular shows promising results in the relief and stabilization of the patients, but its effect is amplified when used in combination with other treatments. More recent studies have focused on evaluating therapeutic potential of CDK4/6 inhibitors when coupled with other drugs in treating CRC. For example, when treating tumors in patient-derived Rb+ colorectal xenograph models, the authors found that a combination of MEK inhibitor Trametinib with Palbociclib significantly reduces tumor volume in comparison to monotherapy. Furthermore, KRAS-mutated cells were especially sensitive to this treatment (Lee et al., 2016; Ziemke et al., 2016). Similiar results were obtained when using a Raf inhibitor (LY3009120) in combination with Abemaciclib, where Ras- and Braf-mutated CRC was especially sensitive to this treatment (Chen et al., 2018). Last, the combination of checkpoint inhibitors like anti-PD1 therapy (SHR-1210) with CDK4/6 inhibitor (SHR6390) is currently evaluated in Phase I and II clinical trial for advanced colorectal cancer (ClinicalTrials.gov Identifier: NCT03601598), but no data have been published yet.

Further studies are necessary for understanding the potential of targeting CDK4/6, together with other genes involved in cell cycle machinery. For example, tumor cells depend on high telomerase activity, which enables them to preserve the telomeres during extensive proliferation. Inducing telomere dysfunctions in tumor cells, using the telomere-specific inhibitor 6-thio-dG, potentiates antitumor responses in mice bearing MC38 tumors (Mender et al., 2020). Therefore, combining CDK4/6 inhibitors for cell cycle arrest and 6-thio-dG might provide a more efficient tumor targeted therapy.

One significant challenge raised by the use of CDK4/6 inhibitors is its effect on normal cells, and especially on the highly proliferating cells, such as activated immune cells found in the tumor microenvironment. Targeting CDKs might disrupt the function of upstream genes involved in the cell cycle, such as sirtuins, in normal cells. Modifications in sirtuin 1 (SIRT1) function are especially important. Even though SIRT1 is also upregulated in the CRC tissue compared to the normal one and it has been linked to tumor size and invasion (Chen et al., 2014; Yu et al., 2016), its function in haematopoiesis is nevertheless crucial (Rimmele et al., 2012). Dysfunctions in SIRT1 in normal cells due to CDK4/6 inhibitor use might therefore potentiate cellular senescence and premature aging in various cellular compartments (Sasaki et al., 2006).

Overall, CDK inhibitors are efficient in preventing colon tumor cells from proliferating by inducing cell cycle arrests, and, in some cases, even apoptosis, making them useful for developing new potential therapeutic strategies for CRC. Nevertheless, a comprehensive analysis on how CDK inhibitors might affect normal cells, as well as the antitumor response of immune cells to CRC, would enhance our understanding on this novel therapy.
